# Upregulation of Cathepsin X in Glioblastoma: Interplay with γ-Enolase and the Effects of Selective Cathepsin X Inhibitors

**DOI:** 10.3390/ijms23031784

**Published:** 2022-02-04

**Authors:** Bernarda Majc, Anamarija Habič, Metka Novak, Ana Rotter, Andrej Porčnik, Jernej Mlakar, Vera Župunski, Urša Pečar Fonović, Damijan Knez, Nace Zidar, Stanislav Gobec, Janko Kos, Tamara Lah Turnšek, Anja Pišlar, Barbara Breznik

**Affiliations:** 1 Department of Genetic Toxicology and Cancer Biology, National Institute of Biology, 111 Večna pot, 1000 Ljubljana, Slovenia; bernarda.majc@nib.si (B.M.); anamarija.habic@nib.si (A.H.); metka.novak@nib.si (M.N.); ana.rotter@nib.si (A.R.); tamara.lah@nib.si (T.L.T.); 2 Jozef Stefan International Postgraduate School, 39 Jamova cesta, 1000 Ljubljana, Slovenia; 3 Department of Neurosurgery, University Medical Centre Ljubljana, 7 Zaloška cesta, 1000 Ljubljana, Slovenia; andrej.porcnik@kclj.si; 4 Institute of Pathology, Faculty of Medicine, University of Ljubljana, 2 Korytkova ulica, 1000 Ljubljana Slovenia; jernej.mlakar@mf.uni-lj.si; 5 Chair of Biochemistry, Faculty of Chemistry and Chemical Technology, University of Ljubljana, 113 Večna pot, 1000 Ljubljana, Slovenia; vera.zupunski@fkkt.uni-lj.si; 6 Faculty of Pharmacy, University of Ljubljana, 7 Aškerčeva cesta, 1000 Ljubljana, Slovenia; ursa.pecarfonovic@ffa.uni-lj.si (U.P.F.); damijan.Knez@ffa.uni-lj.si (D.K.); nace.zidar@ffa.uni-lj.si (N.Z.); stanislav.gobec@ffa.uni-lj.si (S.G.); janko.kos@ffa.uni-lj.si (J.K.)

**Keywords:** glioblastoma, cathepsin X, γ-enolase, tumor microenvironment, glioblastoma stem cells, cathepsin X inhibitors

## Abstract

Glioblastoma (GBM) is the most common and deadly primary brain tumor in adults. Understanding GBM pathobiology and discovering novel therapeutic targets are critical to finding efficient treatments. Upregulation of the lysosomal cysteine carboxypeptidase cathepsin X has been linked to immune dysfunction and neurodegenerative diseases, but its role in cancer and particularly in GBM progression in patients is unknown. In this study, cathepsin X expression and activity were found to be upregulated in human GBM tissues compared to low-grade gliomas and nontumor brain tissues. Cathepsin X was localized in GBM cells as well as in tumor-associated macrophages and microglia. Subsequently, potent irreversible (AMS36) and reversible (Z7) selective cathepsin X inhibitors were tested in vitro. Selective cathepsin X inhibitors decreased the viability of patient-derived GBM cells as well as macrophages and microglia that were cultured in conditioned media of GBM cells. We next examined the expression pattern of neuron-specific enzyme γ-enolase, which is the target of cathepsin X. We found that there was a correlation between high proteolytic activity of cathepsin X and *C*-terminal cleavage of γ-enolase and that cathepsin X and γ-enolase were colocalized in GBM tissues, preferentially in GBM-associated macrophages and microglia. Taken together, our results on patient-derived material suggest that cathepsin X is involved in GBM progression and is a potential target for therapeutic approaches against GBM.

## 1. Introduction

Glioblastoma (GBM) remains the most lethal and common primary brain tumor in adults despite standard treatment including maximal safe surgical removal, chemotherapy, and radiotherapy [1,2]. GBM is classified by the World Health Organization as grade IV glioma and responds poorly to therapy, with a 5-year patient survival rate of less than 5% [3,4,5,6]. Therefore, there is an urgent need to identify new therapeutic targets to develop efficient antitumor approaches.

Inter- and intratumoral heterogeneity [7], presence of therapy-resistant GBM stem cells (GSCs) [8,9], rapid tumor invasion [10], and a supportive tumor microenvironment (TME) [11,12] are responsible for therapy resistance [7,9,11,13,14,15]. GBMs are classified according to The Cancer Genome Atlas (TCGA) into three molecular subtypes based on genomic profiling: proneural (PN), classical (CL), and mesenchymal (MES) GBMs that differ in prognosis and are associated with a unique molecular fingerprint [16,17]. Intratumoral heterogeneity exists at both the genetic and cellular levels. GBM cells co-opt components of the TME to create a complex milieu that promotes tumor development, invasion, and resistance to treatment [12]. Myeloid cells, consisting primarily of two distinct cell types, macrophages and microglia, are greatly affected by the TME [18] and represent the major component of the GBM TME and can account for up to 30–40% of the total tumor mass [19,20] where tumor-associated macrophages (TAMs) are predominant [21,22]. These cells within the tumor mass play a crucial role in enhancing tumor growth and dissemination via suppression of inflammation, promotion of angiogenesis, and extracellular matrix remodeling [18]. 

Cathepsins belong to the C1A papain superfamily of cysteine peptidases, which comprises 11 members, including cathepsins B, L, K, S, and X. Most of the cathepsins are endopeptidases that catalyze the cleavage of peptide bonds within polypeptide chains, whereas some of them are carboxy exopeptidases, such as cathepsins B and X, and amino exopeptidases, such as cathepsins C and H, that cleave their substrates at the C- or N-terminus, respectively [23]. Cathepsins are involved in the regulation of numerous physiological processes and are mostly, but not exclusively, found in endosomes and lysosomes for protein degradation [24] in nonneoplastic human cells. In pathological conditions such as inflammation and cancer, cathepsins may be present in the nucleus, excessively secreted into the cytoplasm and extracellularly, where they can be associated with the plasma membrane of cancer cells [25]. Besides cancer cells, many other noncancerous cell types in the TME express cathepsins, including fibroblasts, neutrophils, mast cells, T cells, endothelial cells, and TAMs, playing specific roles in cancer and antitumor immune response [26]. In tumors, the expression of lysosomal cysteine cathepsins is often elevated and is generally associated with poor patient prognosis [26]. Cathepsins have been shown to exert tumor-promoting functions [27], such as promoting tumor growth, invasion [28], and resistance to therapy [26,29], although some of them also contribute to tumor suppression [30,31,32,33]. 

Not much is known about the expression and function of cathepsin X in GBM. Recently, we have shown that cathepsin X is abundantly expressed in GBM tumors and localizes in perivascular GSC niches, but its role is still unknown [34]. Cathepsin X (also known as cathepsin Z or cathepsin P) is a cysteine carboxypeptidase that is predominantly localized in cells of the immune system such as macrophages, monocytes, and dendritic cells [23], indicating its role in immune cell maturation, proliferation, migration, adhesion, and signal transduction [35,36]. There is increasing evidence that higher levels of cathepsin X are associated with various types of cancer [23] and are presumably involved in the mechanisms of tumor progression that lead to alterations in cellular processes, including cell proliferation and invasion. In addition, cathepsin X has been detected in the brain in glial cells, neurons, oligodendrocytes, and ependymal cells [23,35,37,38,39,40]. Cathepsin X is involved in inflammation-induced neurodegeneration [39,41]. The neurodegenerative action of cathepsin X involves the sequential cleavage of the *C*-terminal amino acids of γ-enolase, abolishing neurotrophic activity of γ-enolase [42]. It is also known as neuron-specific enolase and is an enzyme of the glycolytic pathway expressed predominantly in neurons and cells of the neuroendocrine system. The enzyme occurs as two isozymes, γγ and αγ. The γγ isoform is mainly found in mature neurons, whereas the αγ isoenzyme is localized in nonneuronal cells such as microglia, oligodendrocytes, and astrocytes [43]. Cytoplasmic γ-enolase is involved in enhanced aerobic glycolysis associated with cell proliferation. The additional active site at the *C*-terminal part of the molecule, which is not part of the catalytic element involved in glycolysis, is thought to support the growth, survival, and differentiation of both developing and mature neurons [44,45]. In cancer cells, γ-enolase is considered to act as a pro-survival factor [46]. These pro-survival and neuritogenic activities mediated by γ-enolase are regulated by the cysteine peptidase cathepsin X. However, in brain cancer pathology, the underlying mechanism of γ-enolase action and its regulation by cathepsin X remain unclear.

In the present study, we aimed to investigate the abundance and possible role of cathepsin X in GBM, focusing on the regulation of γ-enolase. Specifically, we investigated the localization as well as the expression level and activity of cathepsin X in tumor tissues from GBM patients. To explore the therapeutic potential of targeting cathepsin X, we tested the effects of its selective irreversible and reversible cathepsin X inhibitors on the viability of patient-derived GBM cells, as well as macrophages and microglia treated with conditioned GBM cell media. To investigate the interplay of cathepsin X and γ-enolase in GBM, colocalization studies were performed with specific antibodies for cathepsin X and γ-enolase in GBM tissue sections. In addition, to reveal the relevance of cathepsin X activity with respect to proteolytic processing of γ-enolase in GBM tissues, the protein levels of the total and the intact active form of γ-enolase were determined in GBM tissue lysates, and the effect of the peptide mimicking the intact *C*-terminal end of γ-enolase on GBM cell, macrophage, and microglia proliferation was assessed.

## 2. Results

### 2.1. Expression and Enzymatic Activity of Cathepsin X Are Upregulated in GBM Tissues 

**Figure 1:**
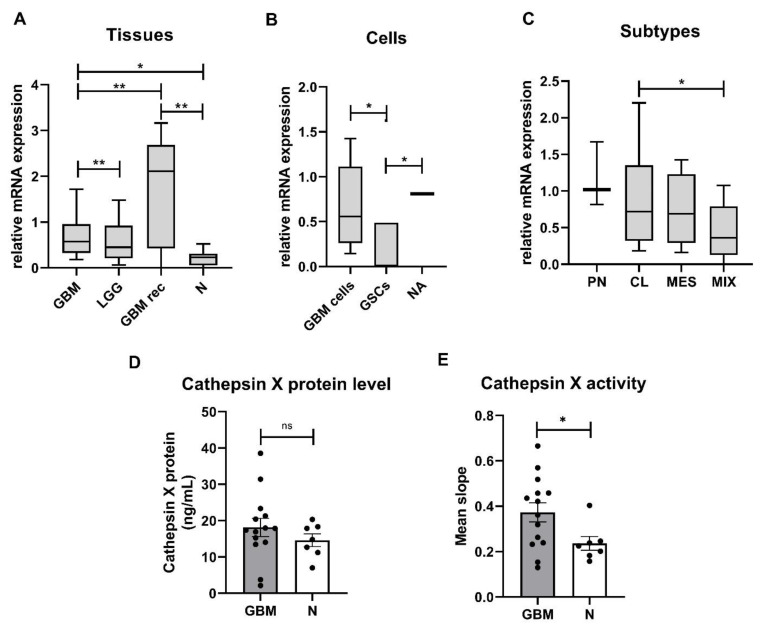
Cathepsin X mRNA levels, protein expression, and enzymatic activity in GBM. (**A**) Relative mRNA level of cathepsin X was increased in de novo GBM tissues (*n* = 43) and recurrent GBM (GBM rec, *n* = 5) compared to LGG (*n* = 14) and nontumor brain tissues (N, *n* = 16). (**B**) Cathepsin X at the mRNA level was expressed in primary GBM cells (*n* = 17) and normal astrocytes (NA, *n* = 1) and at a low level in GSCs (*n* = 6). (**C**) Cathepsin X mRNA was expressed in classical (CL, *n* = 18), proneural (PN, *n* = 3), mesenchymal (MES, *n* = 17), and mixed (MIX, *n* = 25) GBM subtypes. The boxplots show relative cathepsin X mRNA expression in different sample groups. Different Y-axis scales are presented. (**D**) The cathepsin X protein level was not different in GBM tissues (*n* = 14) compared to the nontumor tissue control (N, *n* = 7). (**E**) The cathepsin X enzyme activity was significantly increased in GBM tissues (*n* = 14) compared to the nontumor tissue control (N, n = 7). The data are presented as the mean values ± SEM. * *p* < 0.05, ** *p* < 0.01.

Expression of the cathepsin X gene was evaluated in low-grade gliomas (LGG), GBMs, and nontumor brain tissues as well as in primary GBM cells and GSCs established from GBM tissues. The highest relative mRNA expression of cathepsin X was measured in recurrent GBM, followed by de novo GBM and LGG tissues. The relative mRNA expression of cathepsin X was significantly lower in nontumor brain tissues compared to recurrent and de novo GBM tissues (Figure 1A). Cathepsin X was expressed in GBM cells and normal astrocytes and was higher than in GSCs (Figure 1B). Moreover, we analyzed mRNA levels of cathepsin X in the four GBM subtypes, MES, PN, CL, and mixed (MIX). This classification is based on the expression values of 12 subtype-specific genes according to Behnan et al. [47], to which we added three more genes based on in-house gene expression analyses [48]. The PN subtype was classified by the expression levels of *OLIG2*, *P2RX7, STMN4, SOX10, NOTCH*, and *ERBB3* genes. The CL subtype was classified by the expression levels of *NF-KB, ACSBG1, S100A4*, and *KCNF1*, the MES subtype—by the expression levels of *DAB2, TGFB1, THBS1, COL1A2,* and *COL1A1*. The cathepsin X gene was expressed in all GBM subtypes, but the lowest level was detected in the MIX subtype. Statistical significance was observed only for the relative mRNA expression of cathepsin X between the CL and MIX subtypes (Figure 1C). Cathepsin X median mRNA levels in our study of 48 GBM patients did not correlate with the overall survival of GBM patients (Figure S1). To confirm the differences at the protein level of cathepsin X and its enzymatic activity, proteins were isolated from the dissected GBM and nontumor brain tissues. ELISA revealed no significant difference in cathepsin X expression between the groups; however, we observed a trend towards increased protein level of cathepsin X in GBM (Figure 1D). On the other hand, significantly higher enzymatic activity of cathepsin X (Figure 1E) was detected in GBM tissues as compared to nontumor brain tissues. 

### 2.2. Macrophage-Specific and Microglia-Specific Localization of Cathepsin X in GBM Tissues

**Figure 2:**
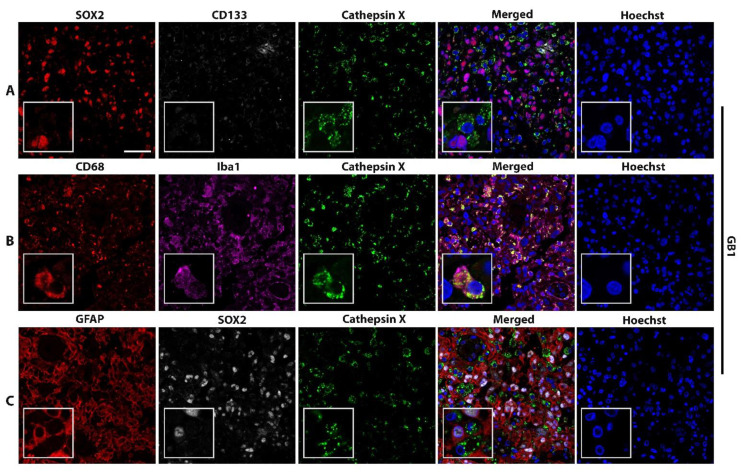
Localization of cathepsin X in CD68-positive and Iba-1-positive cells. Representative images of triple immunofluorescence staining of cathepsin X (green) and markers of **(A)** GSCs (SOX2—red, CD133—grey), **(B)** macrophages and microglia (CD68—red) and microglia (Iba1—purple), and **(C)** GBM cells and astrocytes (GFAP—red) and GSCs (SOX2—grey) are shown. The nuclei were counterstained with Hoechst 33258 (Hoechst, blue). Scale bar = 50 μm.

High levels of the cathepsin X protein were detected in all the tested GBM tissue sections. Next, we analyzed which cells express cathepsin X. As cathepsin X is predominantly expressed in immune-like cells, we used CD68 and Iba1 as biomarkers of immune cells present in GBM tissues, namely macrophages and microglia, respectively. GFAP was used as an astrocytic marker, SOX2 and CD133—as biomarkers for GBM progenitor cells GSCs. Cellular localization of cathepsin X in GBM was determined by immunofluorescence staining in tissue sections from four GBM patients (Figure 2 and Figure S2) and in nontumor brain tissue sections (Figure S2). Figure 2 and Figure S2 show that cathepsin X mostly localized within cells of the innate immune system, macrophages, and microglia (markers CD68, Iba1) and was also expressed in some GFAP-, SOX2-, and CD133-positive cells. Cathepsin X also colocalized with microglia biomarker Iba1 in nontumor brain tissues (Figure S2).

### 2.3. Cathepsin X Inhibition Decreases Viability of Primary Patient-Derived GBM Cells and GBM-Associated Cells

**Figure 3:**
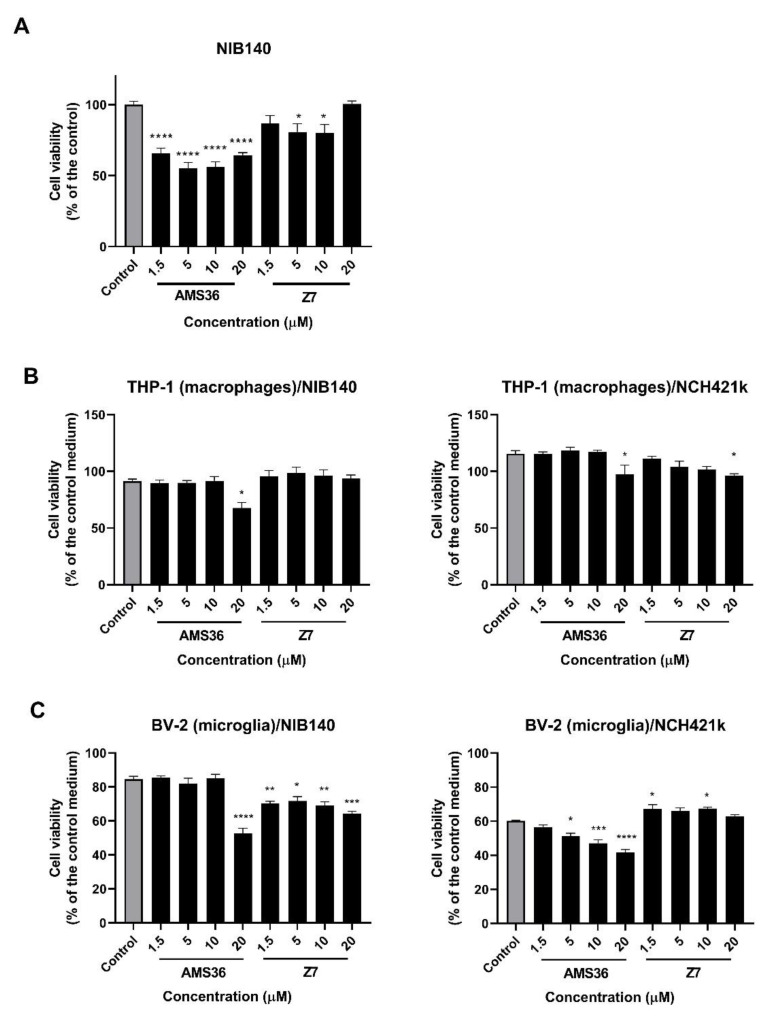
Effects of cathepsin X inhibitors AMS36 and Z7 on viability of GBM cells and GBM-associated cells. GBM cells NIB140 (**A**), differentiated macrophage THP-1 cells (**B**), and BV-2 microglial cells (**C**) exposed to the NIB140- and NCH421k-conditioned media were treated with cathepsin X inhibitors AMS36 and Z7 at various concentrations. Cell viability was then assessed using the MTS assay. The control means a DMSO solvent (0.25%). The control medium was a blank GBM cell/GSC medium without soluble molecules secreted from GBM cells/GSCs. The data are presented as the mean values ± SEM. * *p* < 0.05, ** *p* < 0.01, *** *p* < 0.001, and **** *p* < 0.0001.

First, GBM cell viability and proliferation were examined after 48 h of treatment with two selective inhibitors of cathepsin X, irreversible inhibitor AMS36 and reversible inhibitor Z7. The effect of inhibitors was tested on both primary patient-derived GBM cells NIB140 and GSCs NCH421k. These cells showed differential expression of GBM stem cell and differentiation markers (Figure S3). Primary patient-derived GBM cells NIB140 expressed low levels of stem cell markers and high levels of differentiation markers *GFAP* and *TUB33*. In contrast, GSCs NCH421k expressed higher levels of GSC markers *PROM1, SOX2, OLIG2, NOTCH1, OCT4*, and *CD15* and lower levels of differentiation markers than NIB140 (Figure S3). AMS36 decreased the viability of NIB140 cells up to 45%, of Z7 cells—up to 20% as compared to the vehicle control (0.25% DMSO) (Figure 3A). Similarly, AMS36 and Z7 reduced proliferation of NIB140 cells as shown by the CFSE staining assay. An increase in the CFSE fluorescence intensity of cells means a decrease in cell proliferation in the cell culture. CFSE-labeled NIB140 cells showed an increased mean of CFSE fluorescence intensity of 5% in the presence of AMS36 and Z7 compared to the control (Figure S4A). Proliferation of NCH421k cells was not altered after treatment with cathepsin X inhibitors (Figure S4B). We then tested the effect of cathepsin X inhibitors AMS36 and Z7 on macrophages and microglia that were exposed to soluble factors secreted by GBM cells and GSCs. We performed coculture models where we collected media from GBM cells NIB140 and GSCs NCH421k cells and transferred the conditioned media to PMA-differentiated THP-1 cells (macrophages) and BV-2 cells (microglia) in the presence of increasing concentrations of cathepsin X inhibitors. AMS36 inhibited cell viability of macrophages THP-1 treated with the NIB140/NCH421k-conditioned medium at the highest concentrations tested (Figure 3B). A stronger effect of AMS36 and Z7 on cell viability was observed in microglial BV-2 cells treated with NIB140 and NCH421k conditioned media. AMS36 decreased viability by up to 60% at the highest concentrations (5–20 µM), whereas Z7 also showed an effect at lower concentrations in both BV-2 cells (Figure 3C). To test the effects of cathepsin X inhibitors on GBM cell invasion, a 3D cell invasion assay was performed. All the tested inhibitors inhibited invasion of NIB140 cells by up to 20%. Z7 inhibitor impaired invasion of NCH421k cells by up to 20% (Figure S5A).

### 2.4. Interplay of Cathepsin X and γ-Enolase in GBM Tissues 

**Figure 4:**
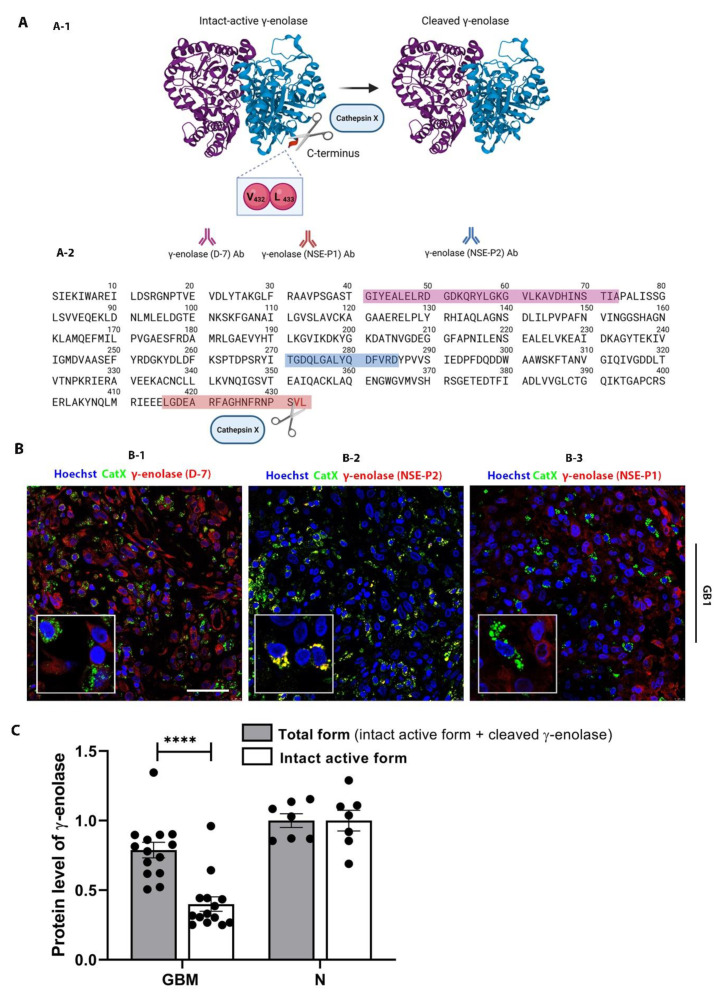
Colocalization of cathepsin X and γ-enolase in GBM tissues. (**A**) A structure of the γ-enolase dimer and cleavage of C-terminal amino acids by cathepsin X (A-1). Three different primary antibodies against γ-enolase were used: A γ-enolase (D-7) antibody specific for the epitope between amino acids 41–73 near the N-terminus (purple amino acid sequence), a γ-enolase (NSE-P2) antibody against the internal region (amino acids 271–285, blue amino acid sequence), and a γ-enolase (NSE-P1) antibody against amino acids 416–433 in the C-terminal region (red amino acid sequence) (A-2). Total γ-enolase was detected using the NSE-P2 antibody, whereas the intact active form was detected using the NSE-P1 antibody. (**B**) Double immunofluorescence staining for cathepsin X and γ-enolase. In Figure B-1 and Figure B-3, no or partial colocalization of cathepsin X and γ-enolase was observed. In Figure B-2, colocalization of both proteins was observed, suggesting that cathepsin X colocalizes mainly with cleaved γ-enolase. The cell nuclei were counterstained with Hoechst 33258 (Hoechst, blue). Scale bar = 50 μm. (**C**) Significantly lower levels of the intact active form of γ-enolase were observed in GBM tissues compared to the nontumor brain tissue control (N). Protein levels of both forms of γ-enolase in GBM and nontumor brain tissues were obtained by means of ELISA. The bars represent the means with individual values (*n* = 14 of GBM and *n* = 7 of nontumor tissue samples) ± SEM. **** *p* < 0.0001. Image A-1 was created with Mol*Viewer [51] based on RCSB PDB (rcsb.org) ID 1TE6 [52] and image A was created with BioRender.com.

**Figure 5:**
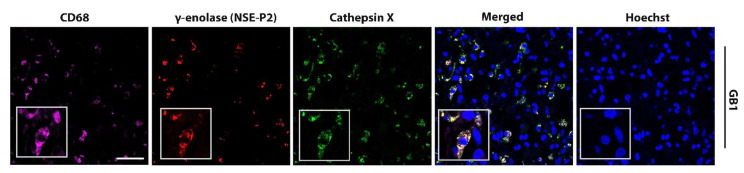
Colocalization of cathepsin X and γ-enolase in CD68-positive cells in GBM tissues. Representative images of triple immunofluorescence staining for cathepsin X (green), γ-enolase NSE-P2 (red), and marker of macrophages and microglia (CD68—purple) show overlapping expression of cathepsin X and γ-enolase NSE-P2 in the CD68-positive cells. The cell nuclei were counterstained with Hoechst 33258 (Hoechst, blue). Scale bar = 50 μm.

Neuron-specific enolase, or γ-enolase, is aberrantly expressed in GBMs [49,50], and it is one of the molecular targets of cathepsin X, which cleaves the C-terminal amino acids L433 and V432 of γ-enolase (Figure 4A-1) [42]. To gain insight into the role of cathepsin X in GBM, we explored the possible interaction of cathepsin X and its target γ-enolase in GBM tissues. To test whether both proteins colocalize, we used three different primary antibodies against γ-enolase: a γ-enolase (D-7) antibody specific for the epitope between amino acids 41–73 near the N-terminus, a γ-enolase (NSE-P2) antibody against the internal region (amino acids 271–285) to detect total γ-enolase, and a γ-enolase (NSE-P1) antibody against amino acids 416–433 in the C-terminal region to detect intact active γ-enolase (Figure 4A and 4B). In Figure 4B-1 and Figure 4B-3, partial or no colocalization of cathepsin X and γ-enolase was observed when using an antibody recognizing the N-terminal (Figure 4B-1) or C-terminal end of γ-enolase (Figure 4B-3). However, in Figure 4B-2 and Figure S6, noticeable colocalization of cathepsin X and γ-enolase was observed when the primary antibodies against the internal region of γ-enolase were used. Because of different colocalization patterns, we next determined the protein levels of the intact active form, which is the C-terminally noncleaved form of γ-enolase, and the total form of γ-enolase in GBM tissues. The protein levels measured by means of “in-house” ELISA were significantly lower for the intact active form of γ-enolase in GBM tissues as compared to total γ-enolase (both the intact active form and cleaved γ-enolase). Additionally, the protein level of noncleaved γ-enolase was significantly lower in GBM tissues as compared to the nontumor brain tissues (Figure 4C). Results of immunofluorescence staining and ELISA suggested that upregulated cathepsin X activity in GBM tissues may regulate the protein level of the intact active end of γ-enolase in GBM by cleaving the C-terminus of γ-enolase (Figure 1E, Figure 4B, and Figure 4C). In order to further explore the type of cells where the colocalization of cathepsin X and γ-enolase is present, we conducted triple immunofluorescence staining to detect colocalization in immune-like cells in GBM tissues. The analysis revealed that cathepsin X and γ-enolase colocalized in CD68-positive cells (Figure 5 and Figure S7).

### 2.5. Proliferation of GBM Cells, GSCs, and GBM-Associated Cells Affected by the γ-Enolase Peptide 

**Figure 6:**
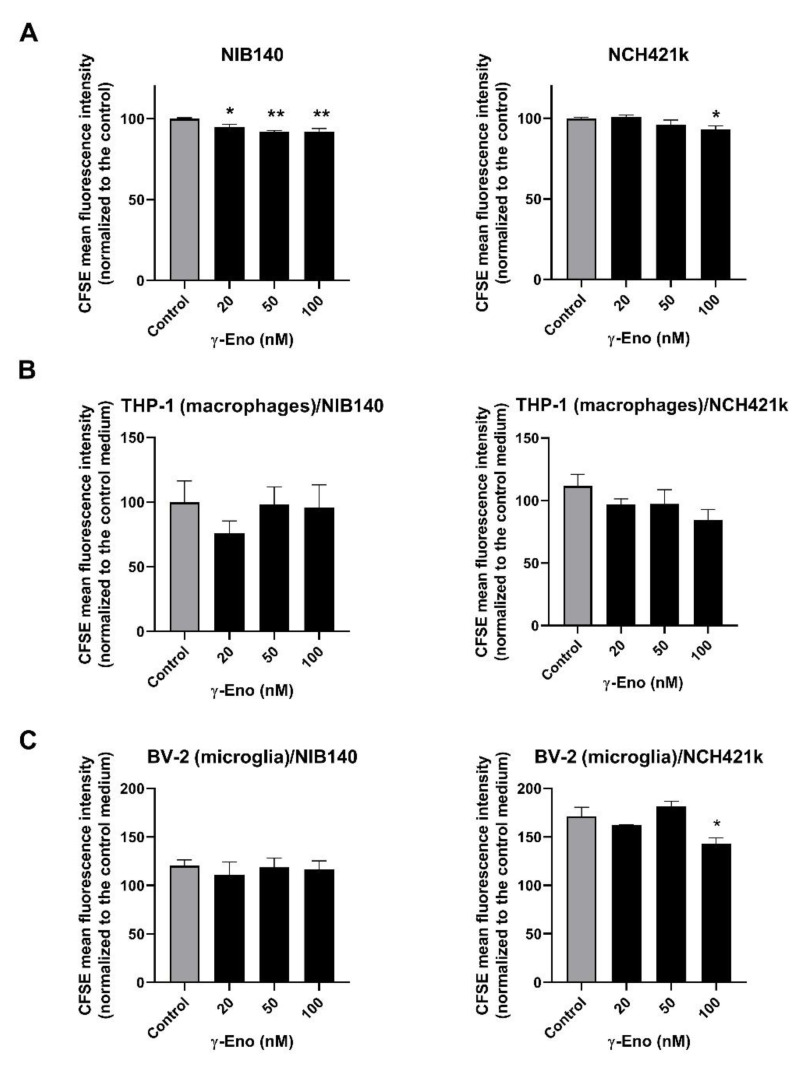
Effects of the γ-Eno peptide on proliferation of GBM cells, GSCs, and GBM-associated cells. GBM cells NIB140, GSCs NCH421k (**A**), as well as differentiated macrophage THP-1 cells (**B**) and BV-2 microglial cells (**C**) exposed to the NIB140- and NCH421k-conditioned media were treated with increasing concentrations of the γ-enolase peptide, C-terminal 30-amino-acid sequence of human brain γ-enolase (γ-Eno). Cell proliferation was evaluated using CFSE staining and flow cytometry. The control means a blank culture GBM cell/GSC medium without addition of γ-Eno (**A**). The control medium is a blank GBM cell/GSC medium without soluble molecules secreted from GBM cells/GSCs and without addition of γ-Eno (**B,C**). The data are presented as the means ± SEM. * *p* < 0.05, ** *p* < 0.01.

As we observed significantly reduced levels of the intact active form of γ-enolase in GBM tissues and as the peptide mimicking the intact *C*-terminal end of γ-enolase promotes proliferation of neuronal cells [44,53], we were interested whether intact active γ-enolase has a functional role for proliferation of cancer cells, macrophages, and microglia. The cells were treated with a range of concentrations of the γ-enolase peptide (γ-Eno), which mimics the *C*-terminal end of γ-enolase, for 48 h, and cell proliferation was evaluated afterwards using CFSE staining. First, proliferation of both GBM cells NIB140 and GSCs NCH421k increased after treatment with the γ-Eno peptide (Figure 6A). The CFSE-stained NIB140 cells showed an up to 8% decrease in the mean fluorescence intensity, and NCH421k cells 7% decrease following γ-Eno treatment. Second, γ-Eno peptide increased the proliferation of microglial BV-2 cells grown in the NCH421k-conditioned media by up to 15%, but did not affect the proliferation of differentiated THP-1 macrophages grown in the NIB140/NCH421k cell-conditioned media (Figure 6B,C). Treatment with the highest concentration of γ-Eno decreased invasion of NIB140 and NCH421k cells by 15% and 25%, respectively (Figure S5B).

## 3. Discussion

We found upregulated levels of cathepsin X in recurrent and de novo GBM tissues compared to less malignant low-grade gliomas and nontumor brain tissues. This is consistent with a previous finding that high expression of cathepsin X correlates with advanced tumor stages in several types of cancer, including prostate, colon, pancreatic, and neuroendocrine cancers [54,55,56,57]. To investigate the association of cathepsin X with GBM intertumoral heterogeneity, we analyzed the gene expression of cathepsin X in four different GBM subtypes and found expression in all the subtypes and no significant differences between the subtypes, except for the lowest expression of cathepsin X in the mixed subtype.

Since increased expression and activity of cathepsin X have been observed in GBM, targeting this enzyme with specific inhibitors opens a new possibility for the treatment of this disease. Several peptidase inhibitors are already in clinical use [58,59], but this is not the case for the inhibitors of cysteine cathepsins. Potent, selective, reversible, and nontoxic inhibitors of cathepsin X were recently developed [60], and one of them, compound Z7, was tested in the present study. In addition, AMS36 was used as an irreversible selective inhibitor of cathepsin X [61,62]. A previous study also suggests that the cathepsin X inhibitor AMS36 can cross the blood–brain barrier. The potency of the cathepsin X inhibitor was determined in male Wistar rats treated with AMS36 at a dose of 50 mg/kg (i.p.). Decreased cathepsin X activity was detected in the cerebellar extracts after two days of AMS36 administration compared with DMSO, indicating that AMS36 crossed the blood–brain barrier [41]. In this study, by selectively inhibiting cathepsin X using these inhibitors, we demonstrated that cathepsin X promotes viability and proliferation of patient-derived GBM cells, but not of GSCs. Moreover, we showed that cathepsin X promotes GBM cell invasion. Invasive growth of residual GBM cells in the surrounding brain tissues is one of the main reasons for the poor prognosis in GBM. Similarly to neural progenitor cells and adult brain stem cells, GBM cells often actively migrate along blood vessels and white matter pathways [63]. Our findings are in line with other studies that showed that cathepsin X is actively involved in cell signaling and promotes cancer cell adhesion, migration, and invasion [23,57,64,65]. Nonetheless, the molecular mechanisms of these effects remain to be elucidated. 

Cathepsin X is expressed in cells of the immune system and in the brain, e.g., in glial cells, neurons, oligodendrocytes, and ependymal cells [23,35,37,38,39,40], in which it cleaves various molecular targets involved in signal transduction, growth, maturation, adhesion, cell–cell communication, proliferation and migration of immune cells. Among nonneoplastic cells, TAMs and microglia have emerged as critical regulators of GBM growth, invasion, angiogenesis, and treatment resistance [12,17,19,21]. We found elevated expression of cathepsin X in GBM cells and of TAMs and microglia in GBM tissues, which is consistent with previous studies in other types of cancer showing that cathepsin X is produced by both cancer cells and TAMs in the TME, influencing cellular crosstalk, cancer proliferation and invasion [57]. As macrophages are found in perivascular GSC niches in GBM [19,21], this may explain the perivascular expression of cathepsin X in our previous study [34]. We showed that inhibition of cathepsin X decreased the viability of macrophages and microglia cultured in conditioned media of GBM cells and GSCs, suggesting the role of cathepsin X in the survival of tumor-supporting TAMs and microglia. By inhibiting cathepsin X, we could not only inhibit GBM cell viability, but also tumor-supporting immune cells such as macrophages and microglia, thereby inhibiting GBM progression. Further studies in more complex tumor models where the immune compartment is maintained, such as organoids or mouse models, are needed to analyze the effects of cathepsin X inhibitors on cancer and immune cells. 

The target of cathepsin X carboxypeptidase activity in brain cells is γ-enolase, which plays an important role in aerobic glycolysis and cell proliferation [45,66,67]; γ-enolase is overexpressed in neurogenic and neuroendocrine tumors [68], and in this study and in others [49], it was shown that it is also expressed in GBM tissues. High levels of γ-enolase are associated with increased aerobic glycolysis, proliferation, and survival of cancer cells [46,49]; γ-enolase also acts as a neurotrophic-like factor and supports growth, survival, and differentiation of neurons [45,66,67]. The intact *C*-terminus of γ-enolase is responsible for the neurotrophic function and is not part of the catalytic element involved in cell glycolysis. It has been shown previously that the peptide mimicking the intact *C*-terminal end of γ-enolase promotes neuronal survival and neurite outgrowth through activation of the phosphatidylinositol 3-kinase/Akt and mitogen-activated protein kinase/extracellular signal-regulated kinase signaling pathways [44]. Similarly, we observed that the γ-Eno peptide mimicking the *C*-terminus of γ-enolase increased the proliferation of GBM cells, GSCs, and GBM-associated immune cells in vitro. 

Cathepsin X cleaves the *C*-terminal dipeptide of γ-enolase, which abolishes its neurotrophic activity [42,44]. In this study, we showed that there is a link between cathepsin X and *C*-terminal cleavage of γ-enolase in the progression of GBM. Namely, cathepsin X activity was upregulated in GBM tissues and correlated with decreased levels of the intact active form of γ-enolase. In comparison, the levels of both forms, intact active and total γ-enolase, were similar in nontumor brain tissues. Moreover, colocalization of cathepsin X with the total form of γ-enolase was observed in GBM tissues, particularly in TAMs and microglia. The latter is consistent with our previous study in which a significantly different colocalization pattern of both forms of γ-enolase was observed in brain tissues. Namely, in a mouse model of Alzheimer’s disease, the C-terminally cleaved form of γ-enolase was associated with cathepsin X in the immediate plaque vicinity, whereas the intact active form was observed in close proximity of the senile plaque, with preferential localization in microglia, which are the resident immune cells in the brain [40]. Nevertheless, the present data suggest that cathepsin X cleaves the *C*-terminal end of γ-enolase in GBM, whereas γ-enolase remains intact in nontumor tissues, where lower cathepsin X protein levels and activity occur.

Cathepsins have been shown to be strong predictive biomarkers for GBM patient survival [27,28,33]. In this study, high levels of cathepsin X do not correlate with survival of GBM patients, although we showed by its selective inhibition that cathepsin X promotes viability of GBM cells and GBM-associated macrophages and microglia as well as proliferation and invasion of GBM cells. This may be due to the fact that cathepsin X can cleave and interact with multiple molecular targets that are involved in different cellular processes [23]. For example, with respect to γ-enolase, cathepsin X cleaves the *C*-terminal amino acids of γ-enolase and presumably neutralizes the proliferative effect of intact active γ-enolase on GBM cells, GSCs, and especially tumor-associated microglia as shown in our study. Thus, cathepsin X also exerts antitumor activity and likely counterbalances tumor-promoting effects.

Taken together, cathepsin X is involved in human GBM progression. Further studies on the molecular mechanisms of cathepsin X and its target γ-enolase are needed to explore its potential for antitumor therapies.

## 4. Materials and Methods

### 4.1. Patient Samples 

Human tissue samples were obtained from patients with LGG (WHO grades I and II) and GBM (WHO grade IV) operated at the Department of Neurosurgery, University Medical Centre Ljubljana, Slovenia. We also obtained 16 tissue samples of nontumor brain tissues. Tumor diagnoses were established using the standard histopathology protocols at the Institute of Pathology of the Medical Faculty, University of Ljubljana. The clinical data and tumor characteristics (histopathological and molecular data) were provided by the Department of Neurosurgery and the Institute of Pathology of the Medical Faculty, University of Ljubljana, Slovenia. The study was approved by the National Medical Ethics Committee of the Republic of Slovenia (approval Nos. 0120-190/2018/23 and 0120-190/2018/4). Written informed consent was obtained from the patients and/or their authorized representatives in accordance with the Declaration of Helsinki.

### 4.2. Establishment of Primary GBM Cells 

To isolate the primary GBM cells, fresh GBM tumor tissue biopsies were minced with scalpels in high-glucose Dulbecco’s modified Eagle’s medium (DMEM) (Hyclone, GE Healthcare, Chicago, IL, USA) supplemented with 10% fetal bovine serum (FBS; Gibco, Thermo Fisher Scientific, Waltham, MA, USA), 2 mM L-glutamine, and 1× penicillin/streptomycin (both: Sigma-Aldrich, St. Louis, MO, USA) and seeded in six-well cell culture plates (Corning, New York, NY, USA). Outgrowing cells were detached with a 0.25% trypsin–EDTA solution (Gibco) and transferred to T25 or T75 cell culture flasks (Corning). The cells were passaged at least three times in this manner and expanded for subsequent analyses. GBM cells were tested using qPCR for expression of GBM cell markers, including CD44, GFAP, and tubulin beta III (TUBB3).

To isolate GSCs, tumor tissue pieces were digested in a digestion buffer (200 U/mL collagenase II and collagenase IV (both: Gibco) in Neurobasal Medium (Invitrogen, Life Technologies, Carlsbad, CA, USA)). Cell suspensions were filtered using a cell strainer with 100 μm pores (BD Falcon, Corning, NY, USA). Single cells were collected and resuspended in complete Neurobasal Medium containing 2 mM L-glutamine, 1× penicillin/streptomycin (both: Sigma-Aldrich), 1× B-27 (Invitrogen), 1 U/mL heparin (Sigma-Aldrich), 20 ng/mL bFGF and EGF (both: Invitrogen). GSCs were cultured as floating spheres in an untreated cell culture flask (Sarstedt Inc., Nümbrecht, Germany). Once these GSC spheres reached 200 μm in diameter, they were dissociated using TrypLE Express (Gibco). GSCs were tested using immunofluorescence and qPCR to express GSC markers, including CD133 (PROM1), SOX2, NOTCH, and OCT4.

### 4.3. GBM and Astrocyte Cell Cultures

GBM stem cell line NCH421k was obtained from CLS (Cell Lines Service GmbH, Eppelheim, Germany). NCH421k cells were grown as floating spheres in complete Neurobasal Medium. All the cell lines were maintained at 37 °C with 5% CO_2_ and 95% humidity. Once these GSC spheres reached 200 μm in diameter, they were dissociated using TrypLE Express (Gibco).

NIB140 cells were the primary patient-derived GBM cells obtained from freshly resected tumor biopsies of the GBM patients operated at University Medical Centre Ljubljana and grown in monolayers in cell culture flasks as described above. All the cell cultures were tested for mycoplasma contamination using a MycoAlert Mycoplasma Detection Kit (Lonza, Basel, Switzerland).

NIB140 and NCH421k cells were tested for gene expression of several stem cell markers, including CD133 (PROM1), SOX2, OLIG2, NOTCH1, OCT4, and CD15, as well as differentiation markers GFAP and TUBB3. Real-time quantitative polymerase chain reaction (RT-qPCR) was used to determine relative mRNA expression of candidate genes (see the next paragraph for details). All the GBM stem cell markers were highly expressed in NCH421k cells and upregulated in comparison to NIB140 cells. NIB140 cells expressed higher levels of differentiation markers GFAP and TUBB3 in comparison to NCH421k cells (Figure S3).

Human astrocytes were purchased from ScienCell Research Laboratories (Carlsbad, CA, USA) and cultured in Astrocyte Medium (ScienCell) supplemented with 2% FBS (ScienCell), 1% astrocyte growth supplement (ScienCell), and 1% penicillin/streptomycin (ScienCell).

### 4.4. Microglia and Macrophage Cell Cultures

Mouse microglial BV-2 cells were a generous gift from Dr. Alba Minelli (University of Perugia, Perugia, Italy). BV-2 cells were cultured in DMEM (Sigma-Aldrich) supplemented with 10% FBS (Gibco), 2 mM L-glutamine, 50 U/mL penicillin, and 50 μg/mL streptomycin (Sigma-Aldrich). The cells were maintained at 37 °C in a humidified atmosphere of 95% air and 5% CO_2_. Confluent cells were subcultured twice or thrice weekly using 0.25% trypsin.

Human THP-1 monocytes were obtained from ATCC (American Type Culture Collection: TIB-202) and grown in suspension in advanced RPMI (Gibco, Thermo Fisher) supplemented with 10% (*v*/*v*) FBS in a humidified, 37 °C, 5% CO_2_ incubator. THP-1 cells were kept at a minimum density of 3 × 10^5^ cells/mL and passaged when reaching 8 × 10^5^ cells/mL. For differentiation, phorbol 12-myristate 13-acetate (PMA) (Sigma-Aldrich) was added to a final concentration of 100 nM. After 48 h, the PMA-supplemented medium was removed, the cells were washed with PBS and treated with GBM and GSC-conditioned media for further analysis.

### 4.5. Coculture Model

To test the effect of soluble factors secreted by GBM, differentiated THP-1 or BV-2 cells were cultured in the complete medium and treated for 48 h with the supernatants of patient-derived GBM cells (NIB140) and GSCs (NCH421k) in the absence or presence of cathepsin X inhibitors AMS36 and Z7 (1.5–20 µM) and the γ-Eno peptide (20–100 nM). After transfer of the GBM cell- and GSC-conditioned media, the cells were examined for cell viability and proliferation index.

### 4.6. Real-Time Quantitative PCR

The tissue samples were snap frozen and stored in liquid nitrogen for further analyses. Total RNA from the tissues and cells was isolated using an AllPrep DNA/RNA/Protein Mini Kit (Qiagen, Germantown, MD, USA) according to the manufacturer’s instructions; cDNA was generated from 1 µg of total RNA of each sample using a High-Capacity cDNA Reverse Transcription Kit (Thermo Fischer Scientific). RT-qPCR was performed to evaluate the mRNA level of genes in our samples. Fluidigm BioMark HD System RT-PCR (Fluidigm Corporation, San Francisco, CA, USA) and 48.48 Dynamic Arrays IFC, where cDNA of 42 samples and 24 TaqMan Gene Expression assays (ThermoFisher Scientific, see Table 1), were mixed pairwise in nanoliter chambers to enable parallel analysis of 2304 reactions. Visualization and analysis of the RT-qPCR results were performed using the Biomark Data Collection software, the Fluidigm RT-qPCR analysis software (both: Fluidigm Corporation), and the quantGenius software [69]. Relative copy numbers of cDNA were normalized to housekeeping genes *HPRT1* and *GAPDH*. Statistical analyses were performed with one-way ANOVA in GraphPad Prism (GraphPad Software Inc., La Jolla, CA, USA).

For PCR analysis, 43 de novo GBM, five recurrent GBM (GBM rec), 14 LGG, 16 nontumor brain (N) tissue samples were analyzed. Moreover, differentiated GBM cells (*n* = 17) and GSCs (*n* = 6) were isolated from GBM tumor biopsies to analyze the relative mRNA expression of cathepsin X. GBM samples and the corresponding clinical data are listed in Table S2.

**Table 1:** List of TaqMan gene expression assays (Thermo Fisher Scientific) used for RT-qPCR.

Gene Name	Assay ID	Assay Type	#
GAPDH	Hs99999905_m1	FAM-MGB, S(250rxns)	4331182
HPRT1	Hs02800695_m1	FAM-MGB, XS(75rxns)	4453320
COL1A2	Hs01028956_m1	FAM-MGB, XS(75rxns)	4331182
COL1A	Hs00164004_m1	FAM-MGB, XS(75rxns)	4331182
TGFBI	Hs00998133_m1	FAM-MGB, XS(75rxns)	4331182
THBS1	Hs00962908_m1	FAM-MGB, XS(75rxns)	4331182
DAB2	Hs01120074_m1	FAM-MGB, XS(75rxns)	4331182
S100A4	Hs00243202_m1	FAM-MGB, XS(75rxns)	4331182
P2RX7	Hs00175721_m1	FAM-MGB, XS(75rxns)	4331182
STMN4	Hs00229288_m1	FAM-MGB, XS(75rxns)	4331182
SOX10	Hs00366918_m1	FAM-MGB, XS(75rxns)	4331182
ERBB3	Hs00176538_m1	FAM-MGB, XS(75rxns)	4331182
ACSBG1	Hs00209500_m1	FAM-MGB, XS(75rxns)	4331182
KCNF1	Hs00266908_s1	FAM-MGB, XS(75rxns)	4331182
OLIG2	Hs00377820_m1	FAM-MGB, XS(75rxns)	4331182
NOTCH1	hs01062014_m1	FAM-MGB, XS(75rxns)	4331182
NFKB1	Hs00765730_m1	FAM-MGB, XS(75rxns)	4331182
CD133 (PROM1)	Hs00195682_m1	FAM-MGB, XS(75rxns)	4331182
SOX2	Hs01053049_s1	FAM-MGB, XS(75rxns)	4331182
OCT4 (POU5F1B)	Hs01596605_s1	FAM-MGB, XS(75rxns)	4331182
CD15 (FUT4)	Hs01106466_s1	FAM-MGB, XS(75rxns)	4331182
GFAP	Hs00909233_m1	FAM-MGB, XS(75rxns)	4331182
TUB33	Hs00801390_s1	FAM-MGB, XS(75rxns)	4331182
Cathepsin X (CTSZ)	Hs00938366_m1	FAM-MGB, XS(75rxns)	4331182

### 4.7. Gene Expression Data Analyses 

#### 4.7.1. GBM Subtyping 

The fifteen selected genes, *COL1A2, COL1A, TGFB1, THBS1, DAB2, S100A4, P2RX7, STMN4, SOX10, ERBB3, ACSBG1, KCBF1, OLIG2, NOTCH1*, and *NFKB1* were used to cluster GBM samples (tissues and cells) into four GBM subtypes: mesenchymal (MES), proneural (PN), classical (CL), and mixed (MIX) [48]. Since the number of subtypes (clusters) was known in advance, we used k-means clustering to partition the expression profiles of the selected genes in one of the four subtypes. The analysis was performed as described in [48] using the R software (version 4.0.3) and its libraries factoextra and cluster [70,71].

#### 4.7.2. Differentially Expressed Genes among the GBM Samples

Differences in the mRNA expression levels of cathepsin X between the GBM samples (tissues and cells) and the previously defined GBM subtypes (mesenchymal—MES, proneural—PN, classical—CL, and MIX—mixed) were analyzed. To minimize the effect of genes with low expression, we first removed them from the analysis by placing the Ct values > 40 as zero. We plotted boxplots to visually assess the differences and variability of the cathepsin X gene expression and then assessed the potential difference between sample types and subtypes using analysis of variance (to determine the homogeneity of variance) followed by Tukey’s post hoc tests. The analyses were conducted in R version 4.0.3.

#### 4.7.3. Survival Analysis

Cox proportional hazards regression was calculated to assess survival in the GBM sample cohorts of different groups. High and low cathepsin X expression groups were determined based on the median expression of cathepsin X. All the analyses were performed in R software version 4.0.3. Logrank test was used to evaluate the statistically significant difference.

### 4.8. Immunofluorescence 

Formalin-fixed and paraffin-embedded tissue sections from six GBM (de novo, WHO grade IV) patients and one noncancerous brain tissue were prepared at the Institute of Pathology and used for immunofluorescence analyses (see Table S1).

The tumor sections were deparaffinized in xylene and rehydrated in ethanol. Heat-mediated antigen retrieval was achieved with a sodium citrate buffer (pH 6.0). Nonspecific binding sites were blocked with a solution of 10% FBS (Gibco; *v*/*v*), 0.1% Triton X-100 (Sigma-Aldrich; *v*/*v*), and 1% BSA (Sigma-Aldrich; *w*/*v*) in 1× PBS (Gibco) for 1 h. After blocking, the sections were incubated with the TrueBlack reagent (Biotium, Fremont, CA, USA) diluted 1:20 in 70% ethanol for 30 s to block autofluorescence due to lipofuscin and blood components. The tissue sections were incubated with the primary antibodies (see Table 2) diluted in 1× PBS containing 1% BSA overnight at 4 °C. After washing with 0.5% BSA in 1× PBS, the tissues were incubated with the secondary antibodies (see Table 3) in 1× PBS containing 1% BSA for 1 h at room temperature. The nuclei were stained with a Hoechst 33258 solution (1:1000; Sigma-Aldrich) for 5 min at room temperature. After washing with 1× PBS, the tissue sections were mounted in a ProLong Gold Antifade mounting solution (Invitrogen), coverslipped, and sealed with nail polish. Confocal imaging was performed using a confocal microscope (SP8 TCS) and the LAS X Life Sciences software (both: Leica, Wetzlar, Germany) at 100× and 200× magnification. Negative control staining was performed in the absence of the primary antibodies.

### 4.9. Protein Extraction from GBM Tissues and Nontumor Brain Tissues

For analysis of the protein levels of cathepsin X and its activity, tissues were homogenized in ice-cold lysis buffer (0.05 M sodium acetate, pH 5.5, 1 mM EDTA, 0.1 M NaCl, 0.25% Triton X-100) supplemented with a cocktail of phosphatase inhibitors (Thermo Fisher Scientific), then sonicated and centrifuged at 15,000× *g* at 4 °C for 15 min to collect the supernatant. Total protein concentration was determined with DC™ Protein Assay (Bio-Rad, Hercules, CA, USA). All the samples were kept at −70 °C until they were used for analysis.

### 4.10. Cathepsin X Activity

Cathepsin X activity was measured in tissue lysates and cell lysates with cathepsin X-specific intramolecularly quenched fluorogenic substrate Abz–Phe–Glu–Lys(Dnp)–OH synthesized by Jiangsu Vcare Pharmatech Co. (China). An aliquot of 50 µg of the lysate proteins was incubated at 37 °C, followed by measurement of cathepsin X activity using 10 µM Abz–Phe–Glu–Lys(Dnp)–OH. The fluorometric reaction was quantified at 37 °C at an excitation wavelength of 320 nm and emission wavelength of 420 nm on a microplate reader (Tecan Safire2). The results are presented as a change in fluorescence as a function of time (ΔF/Δt), and cathepsin X activity was expressed relative to the control.

### 4.11. ELISAs

The protein levels of cathepsin X and γ-enolase in tissue lysates were determined using ELISA as previously reported [35]. For the cathepsin X protein levels, microtiter plates were coated with equal aliquots of goat polyclonal anti-cathepsin X antibody (RD Systems) in 0.01 M carbonate/bicarbonate buffer, pH 9.6, at 4 °C. After blocking with 2% BSA in PBS, pH 7.4, for 1 h at room temperature, the samples with equal protein amounts (50 µg) or cathepsin X standards (0–65 ng/mL) were added. Following 2 h incubation at 37 °C, the wells were washed and filled with a mouse monoclonal anti-cathepsin X 3B10 antibody conjugated with horseradish peroxidase (HRP) in a blocking buffer. Mouse monoclonal 3B10 antibodies were prepared from a mouse hybridoma cell line as reported [35]. After a further 2 h incubation at 37 °C, 200 µL/well of 3,3′,5,5′-tetramethylbenzidine (TMB) substrate (Sigma-Aldrich) in 0.012% H_2_O_2_ was added. After 15 min, the reaction was stopped by adding 50 µL/well of 2 µM H_2_SO_4_. The amount of protein was determined by measuring the absorbance at 450 nm using a microplate reader (Tecan Safire2), and the concentration of cathepsin X was calculated from the standard calibration curve. To measure active γ-enolase, microtiter plates were coated with equal aliquots of the protein in 0.01 M carbonate/bicarbonate buffer, pH 9.6, at 4 °C. After blocking with 2% BSA in PBS, pH 7.4, for 1 h at room temperature, a mouse antibody against *C*-terminal γ-enolase (Santa Cruz Biotechnology) suitable for detecting its active form was added. Following 2 h incubation at 37 °C, the wells were washed and filled with an anti-mouse antibody conjugated with HRP. After further 2 h incubation at 37 °C, 200 µg/well of a TMB substrate in 0.012% H_2_O_2_ was added. After 15 min, the reaction was stopped by adding 50 µL of 2 µM H_2_SO_4_. The amount of cleaved substrate was determined by measuring the absorbance at 450 nm, and the protein levels of cathepsin X and γ-enolase were expressed relative to those in untreated cells (control).

### 4.12. Cathepsin X Inhibitors and γ-Enolase C-Terminal Peptide

The irreversible selective inhibitor of cathepsin X, AMS36, was synthesized according to the modified procedure of Sadaghiani et al. [61] in house [62]. The selective reversible inhibitor Z7 (1-(2,3-dihydrobenzo[b][1,4]dioxin-6-yl)-2-((4-(o-tolyl)-4H-1,2,4-triazol-3-yl)thio)ethan-1-one) was obtained from in-house compound library screening and was synthesized as described [60,61]. The C-terminal 30-amino-acid sequence of human brain γ-enolase (γ-Eno) was synthesized by Biosynthesis (Lewisville, TX, USA), here defined as the γ-enolase peptide (AKYNQLMRIEEELGDEARFAGHNFRNPSVL). The use of the concentration range of cathepsin X inhibitors and the γ-Eno peptide was based on previous studies [40,60,65].

### 4.13. Cell Viability Assay 

MTS [3-(4,5-dimethylthiazol-2-yl)-5-(3-carboxymetoxyphenyl)-2-(4-sulfophenyl)-2H-tetrazolium] colorimetric assay was used to measure viability of GBM cells. NIB140 (5 × 10^3^) and NCH421k (8 × 10^3^) cells were seeded into wells of a 96-well microplate (Thermo Fisher Scientific), and after overnight incubation, they were treated with a range of concentrations of cathepsin X inhibitors (Z7 and AMS36; 0.1–20 µM) and the γ-enolase peptide (20–100 nM) for 48 h. DMSO (final concentration, 0.25%; Sigma-Aldrich) and the culture medium were used as solvent controls for cathepsin X inhibitors and the γ-enolase peptide, respectively. The MTS reagent (Promega, Madison, WI, USA) was then added to the wells of a 96-well microplate and, after incubation, absorbance of formazan was measured at 490 nm on a Synergy Mx microplate reader (Biotek, Winooski, VT, USA). Cell viability (%) was determined as the ratio of absorbance obtained in the presence of the tested compound to that in the solvent alone. Three independent experiments with three replicates per treatment were performed.

### 4.14. Cell Proliferation Assay

A CellTrace Cell Proliferation kit with the CellTrace CFSE reagent (Invitrogen) was used to determine cell proliferation. CFSE fluorescent dye stably incorporates into the cells, and the CFSE content of a cell is divided approximately by half each time the cell divides. By measuring CFSE-labeled cell fluorescence, cell proliferation can be determined. GBM cells were stained with the CellTrace CFSE reagent at a concentration of 1 µM in a cell suspension according to the manufacturer’s protocol. NIB140 (20 × 10^3^) and NCH421k (10 × 10^3^) CFSE-labeled cells were seeded into 24-well culture plates (Corning), respectively. After overnight incubation, the cells were treated with a range of concentrations of cathepsin X inhibitors (Z7 and AMS36; 5–20 µM) and the γ-enolase peptide (20–100 nM) for 48 h. DMSO (final concentration, 0.25%; Sigma-Aldrich) and the culture medium were used as solvent controls for cathepsin X inhibitors and the γ-enolase peptide, respectively. Temozolomide (TMZ; Sigma-Aldrich) in a concentration of 100 µM was used as the positive control. The cells were harvested using TrypLE Express (Gibco), and the mean fluorescence intensities of the cells were measured in the B1 channel using a MACSQuant Analyzer 10 flow cytometer and MACSQuantify Software V3 (both: Miltenyi Biotec, Bergisch Gladbach, Germany). The obtained data were analyzed in FlowJo software V10 (Becton Dickinson, Franklin Lakes, NJ, USA). The mean fluorescence intensity of CFSE reagent staining was normalized to the solvent control. Three independent experiments with two replicates per treatment were performed.

### 4.15. Statistical Analyses

Tukey’s post hoc test, one-way ANOVA test with Dunnett’s multiple comparison, unpaired t-test with Welch‘s t-correction, and multiple t-test followed by a two-stage linear step-up procedure of the Benjamini, Krieger, and Yekutieli correction were used to perform statistical analyses in GraphPad Prism software (GraphPad Software Inc., La Jolla, CA, USA) version 8. *p*-values < 0.05 were considered to indicate significant differences. The *p*-values were expressed within the Figures as follows: **** *p* < 0.0001, *** *p* < 0.001, ** *p* = 0.001–0.01, * *p* = 0.01–0.05.

## Figures and Tables

**Table 2:** List of the primary antibodies.

Primary Antibodies	Source	Dilution
Goat polyclonal anti-cathepsin X	R&D System (AG934)	1:200
Mouse monoclonal anti-Iba1	Abcam (ab15690)	1:200
Rabbit polyclonal anti-CD68	Atlas antibodies (HPA048982)	1:2500
Mouse monoclonal anti-SOX2	Abcam (ab171380)	1:50
Rabbit polyclonal anti-CD133	Abcam (ab19898)	1:100
Rabbit polyclonal anti-GFAP	Abcam (ab211271)	1:1000
Mouse monoclonal anti-γ-enolase (NSE-P1)	Santa Cruz Biotechnology (sc-21738)	1:250
Mouse monoclonal anti-γ-enolase (NSE-P2)	Santa Cruz Biotechnology (sc-21737)	1:250
Mouse monoclonal anti-γ-enolase (D-7)	Santa Cruz Biotechnology (sc-376375)	1:250

**Table 3:** List of the secondary antibodies.

Secondary Antibodies	Source	Dilution
Donkey anti-goat IgG (H+L) Highly Cross-Adsorbed Secondary Antibody, Alexa Fluor Plus 488	Thermo Fisher Scientific (A32814)	1:200
Donkey anti-mouse IgG (H+L) Highly Cross-Adsorbed Secondary Antibody, Alexa Fluor Plus 647	Thermo Fisher Scientific (A32787)	1:200
Donkey anti-rabbit IgG (H+L) Highly Cross-Adsorbed Secondary Antibody, Alexa Fluor Plus 546	Thermo Fisher Scientific (A10040)	1:200
Donkey anti-mouse IgG (H+L) Highly Cross-Adsorbed Secondary Antibody, Alexa Fluor Plus 546	Thermo Fisher Scientific (A10036)	1:200

## Data Availability

All the data supporting the reported results can be provided upon request to the corresponding authors.

## Supplementary Materials

The following supporting information can be downloaded at https://www.mdpi.com/article/10.3390/ijms23031784/s1. References [72,73,74,75] are cited in Supplementary Method 1.
