# Supplementary material for: Upregulation of Cathepsin X in Glioblastoma: Interplay with γ-Enolase and the Effects of Selective Cathepsin X Inhibitors
Source: Int J Mol Sci. 2022 Feb 4;23(3):1784. doi: 10.3390/ijms23031784 (PMC8836869; doi:10.3390/ijms23031784)
Supplement: Supplementary file 1 [file ijms-23-01784-s001.zip › ijms-1568715-supplementary.pdf]

Supplementary information

Upregulation of Cathepsin X in Glioblastoma: Interplay with γ-Enolase and the Effects of Selective Cathepsin X Inhibitors

Bernarda Majc [1,2], Anamarija Habič [1,2], Metka Novak [1], Ana Rotter [1], Andrej Porčnik [3], Jernej Mlakar [4], Vera Županski [5], Urša Pečar Fonović [6], Damijan Knez [6], Nace Zidar [6], Stanislav Gobec [6], Janko Kos [6], Tamara Lah Turnšek [1,5], Anja Pišlar [6,*] and Barbara Breznik [1,*]

[1] Department of Genetic Toxicology and Cancer Biology, National Institute of Biology, 111 Večna Pot, 1000 Ljubljana, Slovenia; bernarda.majc@nib.si (B.M.); anamarija.habic@nib.si (A.H.); metka.novak@nib.si (M.N.); ana.rotter@nib.si (A.R.); tamara.lah@nib.si (T.L.T)

[2] Jozef Stefan International Postgraduate School, 39 Jamova Cesta, 1000 Ljubljana, Slovenia

[3] Department of Neurosurgery, University Medical Centre Ljubljana, 7 Zaloška Cesta, 1000 Ljubljana, Slovenia; andrej.porcnik@kclj.si

[4] Institute of Pathology, Faculty of Medicine, University of Ljubljana, 2 Korytkova Ulica, 1000 Ljubljana Slovenia; jernej.mlakar@mf.uni-lj.si

[5] Chair of Biochemistry, Faculty of Chemistry and Chemical Technology, University of Ljubljana, 113 Večna Pot, 1000 Ljubljana, Slovenia; vera.zupunski@fkkt.uni-lj.si

[6] Faculty of Pharmacy, University of Ljubljana, 7 Aškerčeva Cesta, 1000 Ljubljana, Slovenia; ursa.pecarfonovic@ffa.uni-lj.si (U.P.F.); damijan.Knez@ffa.uni-lj.si (D.K.); nace.zidar@ffa.uni-lj.si (N.Z.); stanislav.gobec@ffa.uni-lj.si (S.G.); janko.kos@ffa.uni-lj.si (J.K.)

* Correspondence: barbara.breznik@nib.si (B.B.); anja.pislar@ffa.uni-lj.si (A.P.); Tel.: +386-(0)59-232-870 (B.B.); +386-(0)14-169-526 (A.P.)

1. Supplementary Methods

3. D Invasion Assay

3D invasion assay was performed as described previously [73–75]. Briefly, tumor spheroids of 5 × 10^3 cells were prepared in U-bottom 96-well plates using 4 % methylcellulose and centrifugation to obtain 1 spheroid per well [75]. After their formation, tumor spheroids were embedded in Matrigel (Corning; 5 mg/mL) and treated with cathepsin X inhibitors and γ-Eno peptide or respective solvent controls (DMSO or culture medium). Invading spheroids of NIB140 and NCH421k cells were imaged after 4 and 7 days of incubation, respectively, using an inverted fluorescence microscope Eclipse Ti (Nikon, Tokyo, Japan) at 4× magnification. Invasive area of spheroids and spheroid diameter were measured using ImageJ software (NIH). Invasive area was normalized to spheroid diameter [76]. Relative invasive area was determined as a ratio of invasive area obtained in the presence of compounds tested to that of solvent alone. Three independent experiments with six replicates per treatment were performed.

2. Supplementary Figures

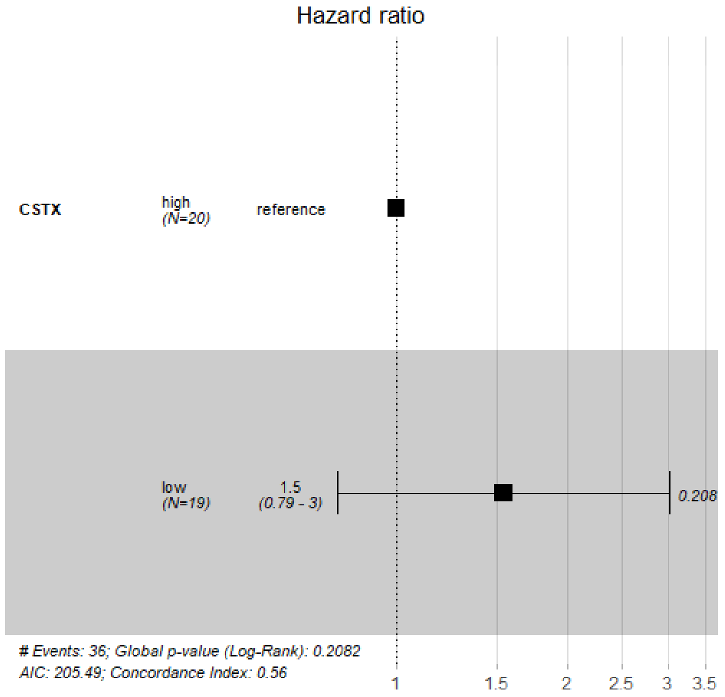

Figure S1. Cathepsin X relative mRNA levels were not associated with the overall survival of GBM patients. Forest plot model showing the association between cathepsin X mRNA levels (low and high) and overall survival of GBM patients with confidence interval of 95% CI. Cox proportional hazard regression was calculated to assess survival in GBM sample cohorts of different groups. Log-rank test was used to evaluate statistically significant differences.

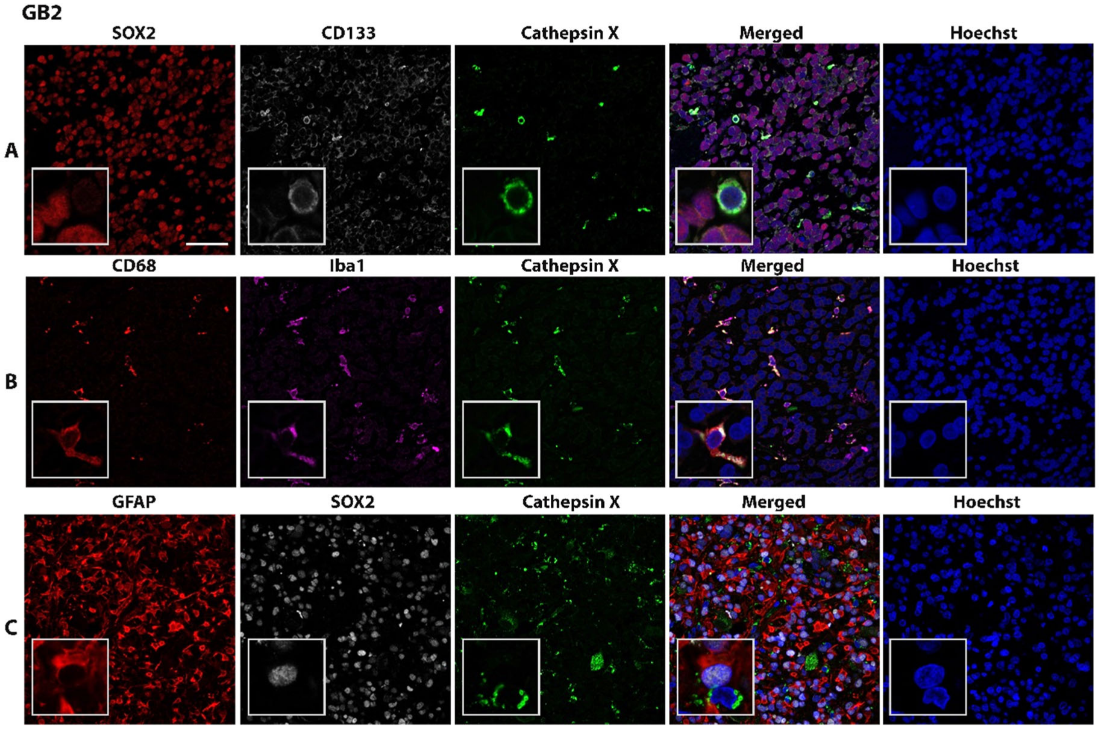
GB2
SOX2
CD133
Cathepsin X
Merged
Hoechst
A
CD68
Iba1
Cathepsin X
Merged
Hoechst
B
GFAP
SOX2
Cathepsin X
Merged
Hoechst
C

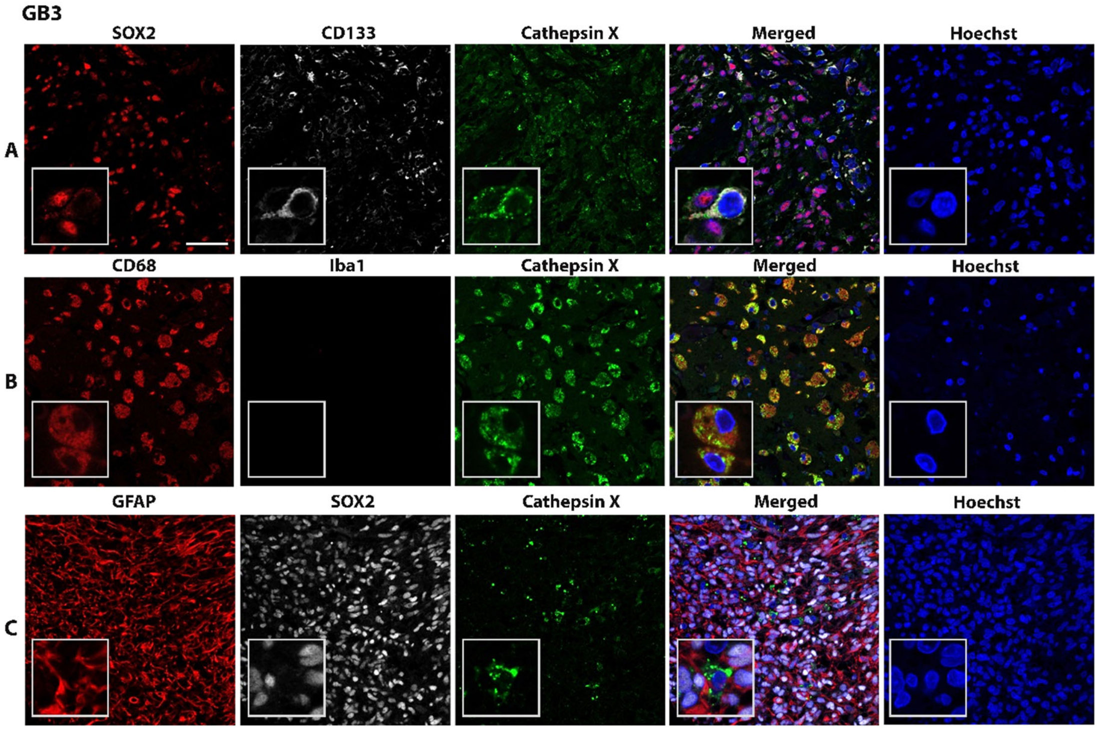
GB3
SOX2
CD133
Cathepsin X
Merged
Hoechst
A
CD68
Iba1
Cathepsin X
Merged
Hoechst
B
GFAP
SOX2
Cathepsin X
Merged
Hoechst
C

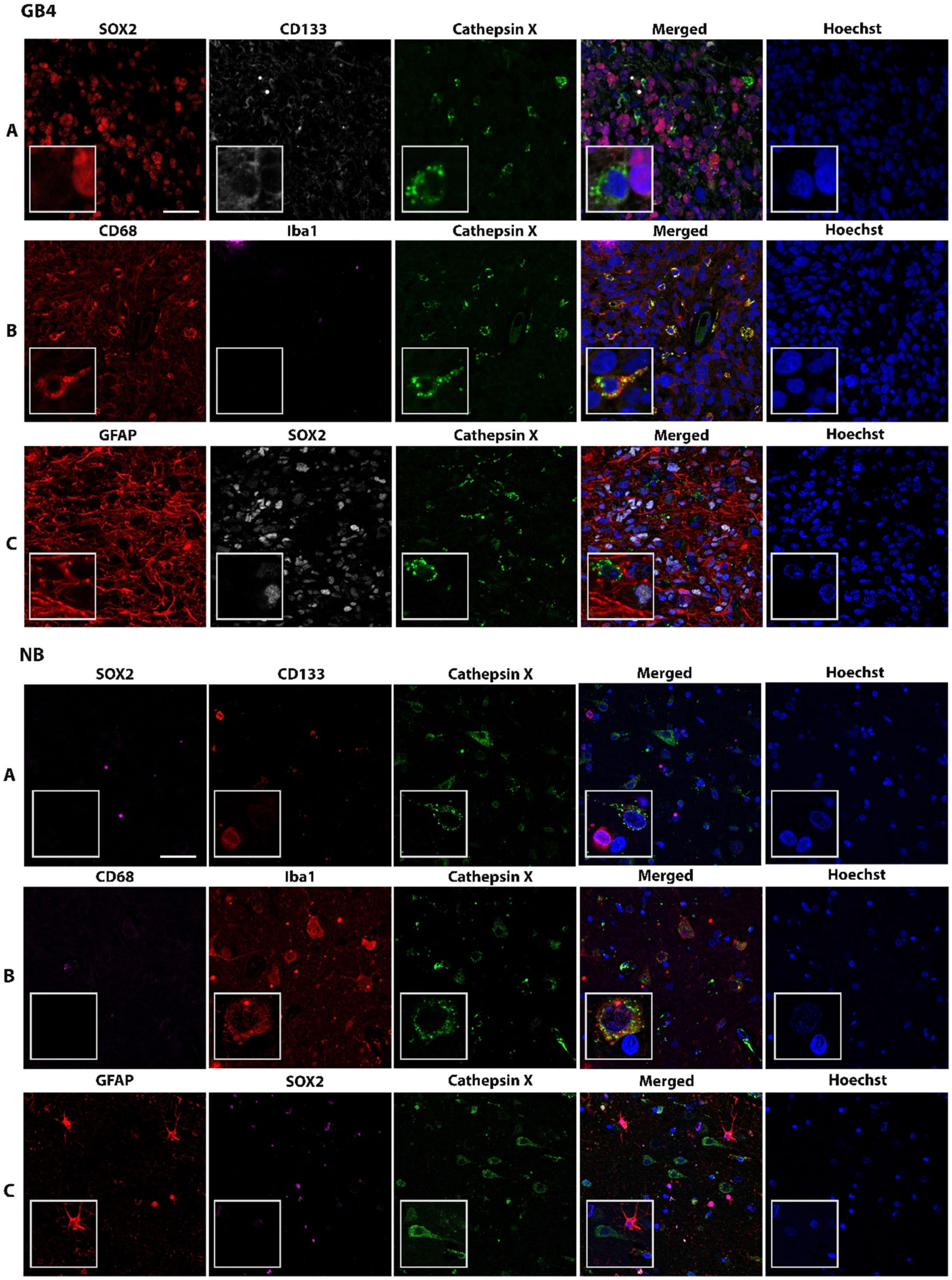

Figure S2. Expression and cell-specific localization of cathepsin X in macrophages and microglia in GBM tissues, in relation to Figure 2. Images of triple immunofluorescence staining of cathepsin X (green) and markers of (**A**) GSCs (SOX2-red, CD133-grey), (**B**) macrophages and microglia (CD68-red) and microglia (Iba1-purple), and (**C**) GBM cells and astrocytes (GFAP-red) and GSCs (SOX2-grey) for three additional GBM patient samples and non-tumor brain tissue (NB1) as control are shown. Scale bar = 50 μm.

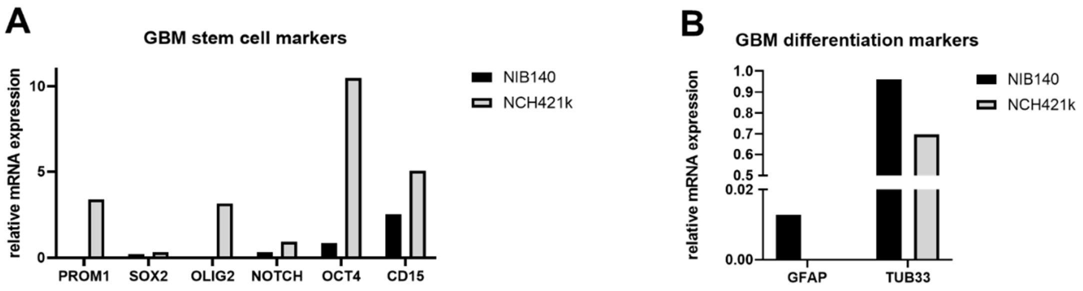

Figure S3. mRNA levels of several GSC (A) and differentiation markers (B) in NIB140 GBM cells and NCH421k GSCs. mRNA expression of markers PROM1, SOX2, OLIG2, NOTCH1, OCT4, CD15, GFAP and TUB33 were determined by RT-qPCR and normalized to housekeeping genes HPRT1 and GAPDH and analyzed with quantGenius software.

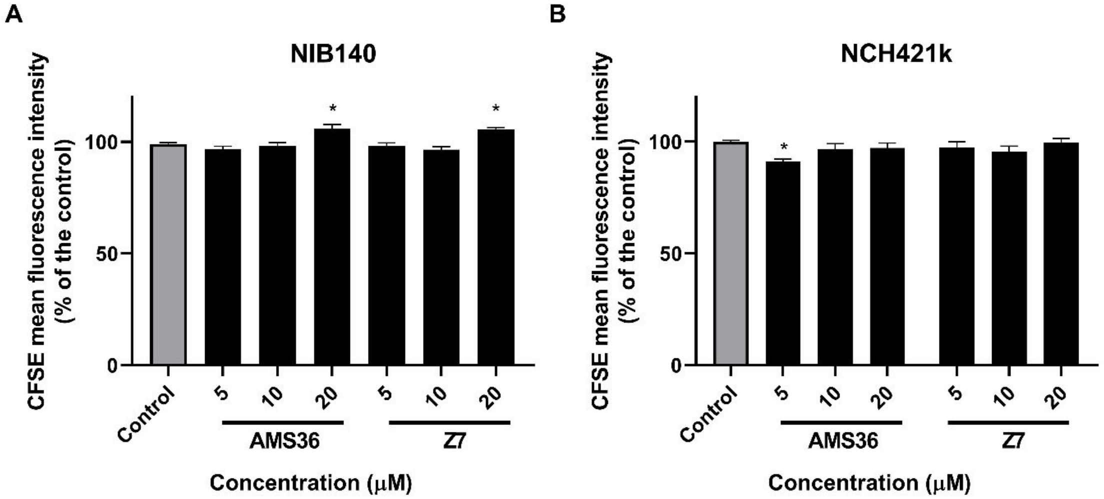

Figure S4. Effects of cathepsin X inhibitors AMS36 and Z7 on proliferation of NIB140 GBM cells and NCH421k GSCs. NIB140 and NCH421k cells were treated with cathepsin X inhibitors AMS36 and Z7 at different concentrations. Cell proliferation was assessed by CFSE staining and flow cytometry. Proliferation of cells was not altered after treatment with cathepsin X inhibitors, or was only affected at the lowest concentration of the AMS36 inhibitor. Control means solvent DMSO (0.25%). Data are presented as mean values ± S.E.M. Statistical analyses were performed with GraphPad Prism software using the the one-way ANOVA – Dunnett’s multiple comparisons test (* $p < 0.05$).

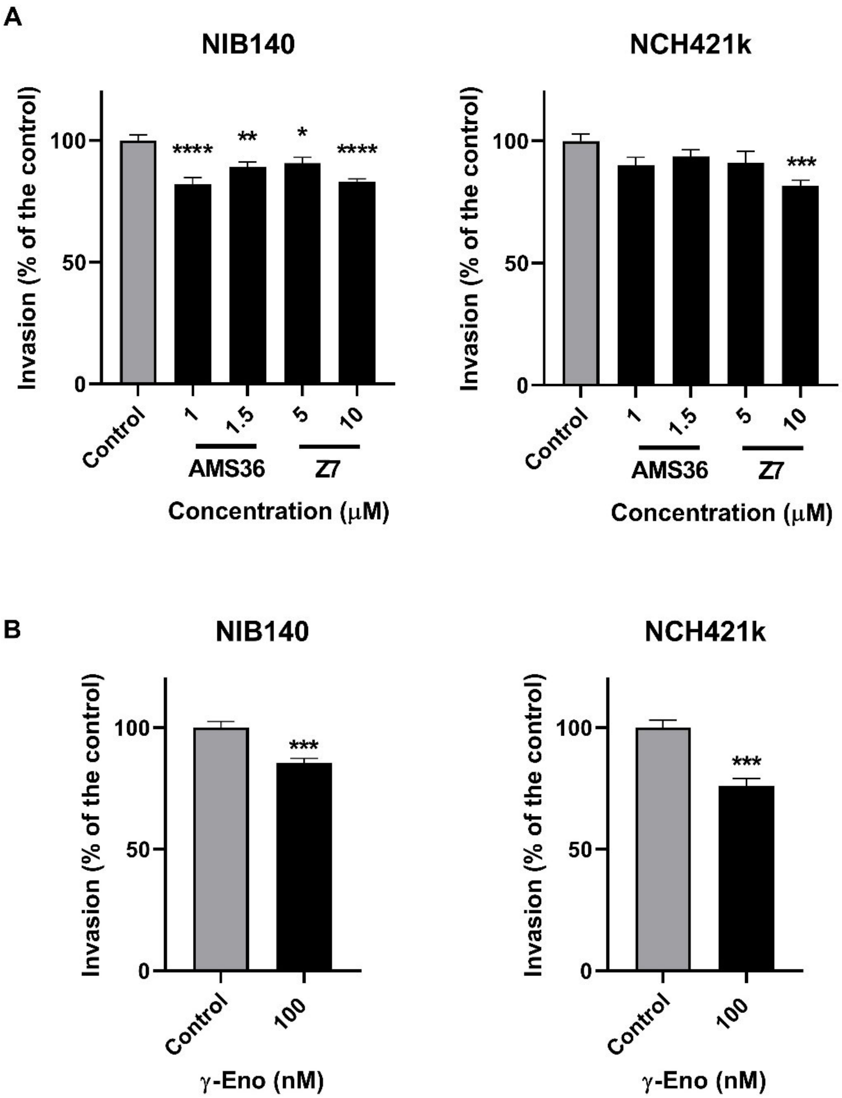

Figure S5. Effects of cathepsin X inhibitors and γ-enolase peptide (γ-Eno) on invasion of GBM cells and GSCs. **(A)** All tested inhibitors inhibited invasion of NIB140 cells up to 20%. Z7 inhibitor impaired invasion of NCH421k cells up to 20%. **(B)** Treatment with the highest concentration of γ-Eno peptide decreased invasion of NIB140 and NCH421k cells for 15% and 25%, respectively. Control means solvent DMSO (0.25%) in **(A)** and blank GBM cell/GSC medium without addition of γ-Eno peptide in **(B)**. Control is solvent DMSO (0.25%). Data are presented as mean values ± S.E.M. Statistical analyses were performed with GraphPad Prism software using the one-way ANOVA – Dunnett's multiple comparisons test (* $p < 0.05$, ** $p < 0.01$, and *** $p < 0.001$).

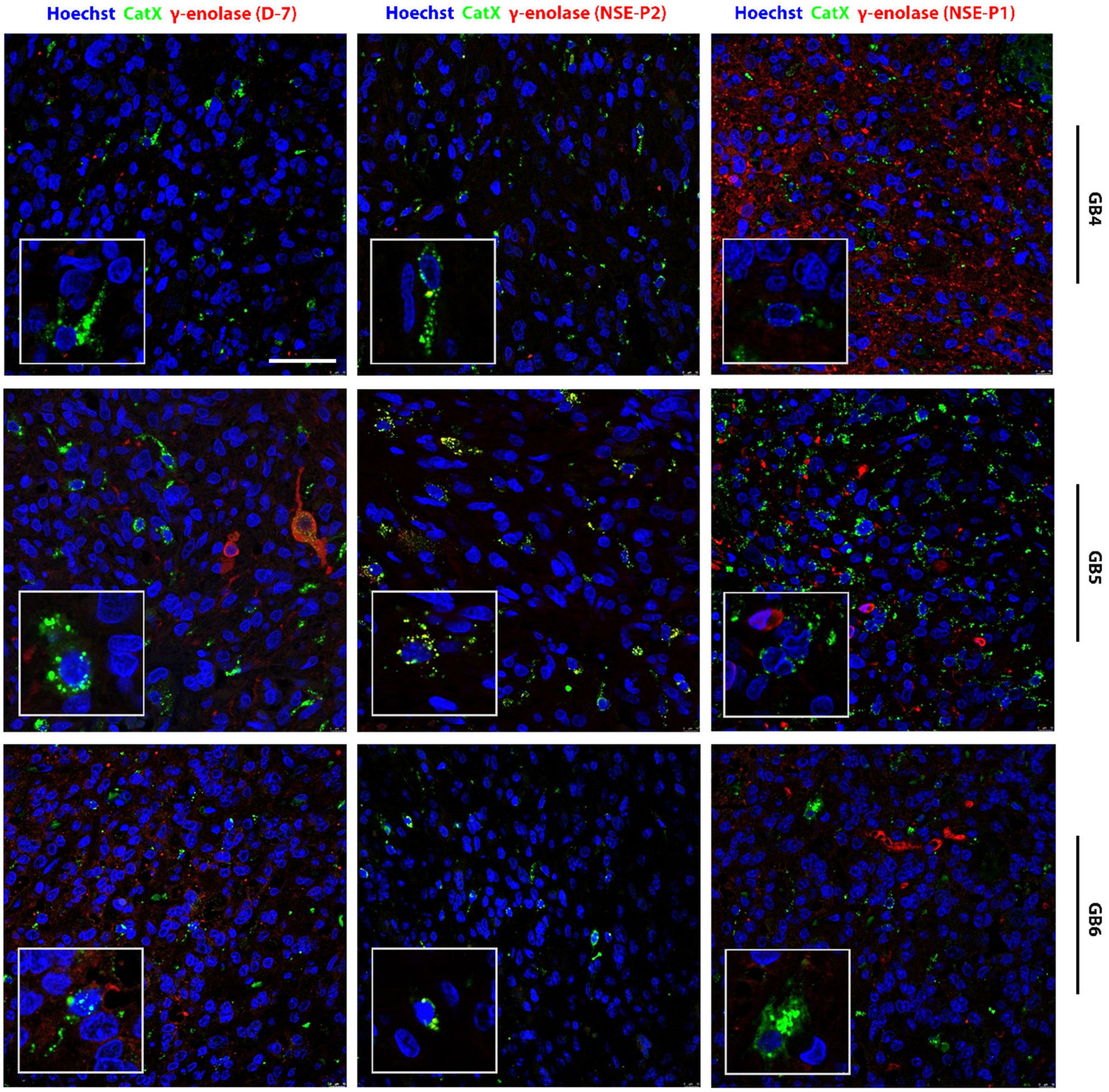

Figure S6. Co-localization of cathepsin X and γ-enolase in GBM tissue sections, in relation to Figure 4B. Images of triple immunofluorescence staining of cathepsin X and γ-enolase are shown with three different antibodies against γ-enolase for three additional tumor samples. Co-localization was observed only when using the antibody against the internal region (NSE-P2). Scale bar = 50 μm.

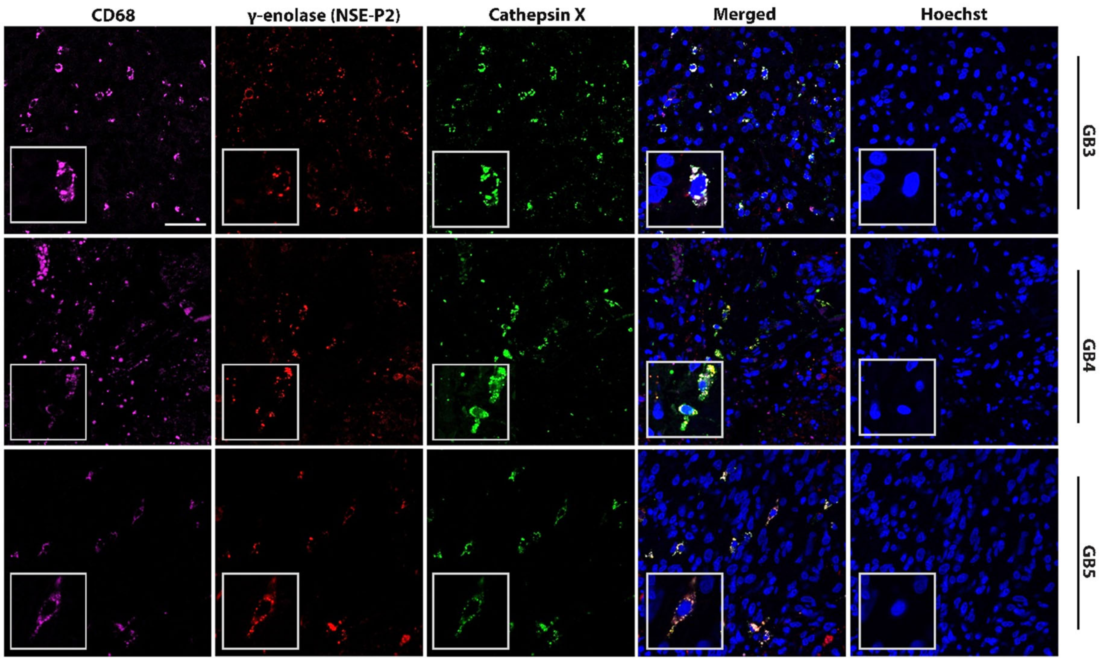

Figure S7. Co-localization of cathepsin X and γ-enolase (NSE-P2) in CD68-positive cells, in relation to Figure 5. Triple immunofluorescence staining of cathepsin X (green), γ-enolase (red), and marker of macrophages and microglia (CD68-purple) shows overlapping expression of cathepsin X and γ-enolase NSE-P1 in macrophages. Scale bar = 50 μm.

3. Supplementary Tables

Table S1. Details of the 6 GBM patients and their tumors operated at the Department Neurosurgery of the University Medical Centre Ljubljana, Slovenia, used for ELISA, cathepsin X activity assessment and immunofluorescence staining.

Patient	Gender	Age (Years)	MGMT Promoter Methylation	Mutations				Chromosomal Abnormalities
				IDH1	TP53	EGFR mutation/ amplification	Other	
**GB1**	M	47	no	wt	mut (P190L)	mut (R108K) / ampl	CDKN2A and CDKN2B del / pTERT (C228T) / MET, PIK3CA ampl / PTEN (I122F)	10q del
**GB2**	M	63	yes	mut (R132H)	mut (V157G)	wt	CDKN2A and CDKN2B del / ATRX (Y2083N) / PIK3R1 (D464del)	chromosome 7 ampl
**GB3**	M	76	yes	wt	wt	ampl	CDKN2A and CDKN2B del / PTEN (G132S), MDM4 (K374G), PDGFRA (G79D)	chromosome 7 ampl / 10q, 19q del
**GB4**	M	79	yes	wt	Splice site mutation / mut (P190L)	ampl	CDKN2A and CDKN2B del / PTEN (C136R), TERT (C250T) / MET, MYCN, PDGFRA ampl	chromosome 7 ampl / 10q del / 1p part del
**GB5**	M	64	no	wt	mut (C238Y)	wt	TERT (C228T)	chromosome 7 ampl / 10q del
**GB6**	M	74	no	wt	mut (P190L)	ampl	CDKN2A and CDKN2B del / TERT (C228), ERBB3 (G337Q)	chromosome 7 ampl / 10q, 13q, 22q del / 14q part del

Abbreviations: mut, mutated; del, deletion; part del, partial deletion; ampl, amplification; wt, wild type.

Table S2. Details of the 43 GBM, 5 GBM rec, 14 LGG patients and their tumors operated at the Department Neurosurgery of the University Medical Centre Ljubljana, Slovenia, used for real-time quantitative PCR analysist, ELISA and cathepsin X activity assessment.

Patient	Gender	Age (Years)	MGMT Promoter Methylation	Mutations				Chromosomal Abnormalities	Survival (Months)
				IDH1	TP53	EGFR Mutation/ Amplification	Other		
GB7	F	69	N.A.	N.A.	N.A.	N.A.	N.A.	N.A.	3
GB8	M	49	N.A.	-	-	N.A.	N.A.	N.A.	8
GB9	F	79	N.A.	-	-	N.A.	N.A.	1p del	4
GB10	F	64	N.A.	-	+	N.A.	N.A.	N.A.	4
GB11	M	70	N.A.	-	+	N.A.	N.A.	N.A.	9
GB12	F	81	N.A.	-	-	N.A.	N.A.	N.A.	6
GB13	F	58	N.A.	-	-	N.A.	N.A.	N.A.	50
GB14	F	66	N.A.	-	-	N.A.	N.A.	N.A.	3
GB15	M	81	N.A.	-	+	N.A.	N.A.	N.A.	6
GB16	F	67	N.A.	-	-	N.A.	ATRX mut	N.A.	2
GB17	M	71	N.A.	-	-	N.A.	N.A.	N.A.	12
GB18	M	58	N.A.	-	-	c.866C>T, p.Ala289Val (p.A289V)	N.A.	N.A.	0
GB19	M	53	N.A.	-	-	part of exons 2-7	N.A.	N.A.	33
GB20	F	59	N.A.	-	+	-	-	N.A.	6
GB21	M	61	N.A.	-	-	N.A.	N.A.	Aneuplodia (35% of cells)	2
GB22	M	62	N.A.	-	-	N.A.	N.A.	N.A.	22
GB23	M	51	N.A.	-	-	N.A.	N.A.	N.A.	0
GB24	M	65	N.A.	-	-	N.A.	N.A.	N.A.	6
GB25	F	84	N.A.	-	Dubious result	N.A.	-	N.A.	7
GB26	M	65	N.A.	-	+	N.A.	KRAS (p.Gly13Asp (G13D) and (kol 33) / CDK4 (kol 36) / NMYC ampl (kol 49)	N.A.	7
GB27	M	73	N.A.	-	-	wt	-	-	5
GB28	M	76	N.A.	-	-	wt	MET (c.3029C>T, p.Thr1010Ile) / PIK3CA (p.Glu542Lys (E542K))	N.A.	3
GB29	F	65	N.A.	-	-	EGFR ampl (kol. 76)	p.Ala289Val (A289V)	-	11
GB30	M	70	N.A.	-	-	EGFR ampl (kol. 85), del eks 2-7 (EG-FRvIII)	-	N.A.	2
GB31	F	48	N.A.	-	-	N.A.	N.A.	N.A.	7
GB32	F	48	N.A.	N.A.	-	N.A.	-	N.A.	48
GB33	F	63	N.A.	-	-	-	-	N.A.	18
GB34	M	56	N.A.	-	+	EGFR ampl (kol. 54), part of exons. 2-7 EGFR	KIT ampl (kol.9) / PDGFRA ampl (kol.9)	N.A.	2
GB35	M	60	N.A.	-	-	EGFR ampl (kol. 45), del. eks. 2-7	-	N.A.	12
GB36	M	71	N.A.	-	-	EGFR ampl (kol. 41)	CDK4 ampl (kol. 49)	N.A.	2
GB37	M	77	yes	-	-	-	-	N.A.	9
GB38	M	56	N.A.	N.A.	N.A.	N.A.	-	N.A.	1

GB39	M	50	N.A.	-	-	EGFR ampl (kol. 106), del. eks. 2-7 EGFR	-	N.A.	22
GB40	M	80	yes	-	-	-	-	N.A.	22
GB41	M	34	N.A.	-	-	-	KRAS mut c.34G>C, p.(Gly12Arg) (G12R) in exn 2, KRAS amp (kol 14)	N.A.	28
GB42	F	69	yes	-	-	EGFR ampl (kol. 44), del. eks. 2-7 EGFR	-	N.A.	14
GB43	F	73	yes	-	-	EGFR ampl (kol. 34), del. eks. 2-7 EGFR	-	N.A.	4
GB rec	F	40	yes	-	-	part of exons 2-7 in EGFR (EGFRvIII) ATRX mut	ATRX mut	-	25
GB rec	F	58	N.A.	-	-	N.A.	N.A.	N.A.	22
GB rec	F	72	N.A.	-	-	part of exons 2-7 in EGFR (EGFRvIII) ATRX mut	N.A.	N.A.	20
GB rec	M	63	N.A.	-	+	ATRX mut	BRAF V600E	-	112
GB rec	M	65	N.A.	-	-	ATRX mut	N.A.	N.A.	16
LGG 1	M	33	N.A.	-	+	N.A.	N.A.	-	22
LGG 2	M	25	N.A.	+	-	N.A.	-	-	61
LGG 3	M	29	N.A.	+	-	N.A.	N.A.	-	102
LGG 4	M	36	N.A.	+	-	N.A.	-	-	41
LGG 5	F	57	N.A.	+	-	N.A.	-	Co-deletion 1p/19q	39
LGG 6	M	66	N.A.	+	-	N.A.	-	N.A.	162
LGG 7	M	73	N.A.	IDH1 R132H	Dubious result	N.A.	ATRX mut	N.A.	51
LGG 8	F	61	N.A.	+	+	N.A.	ATRX mut	-	144
LGG 9	M	44	N.A.	+	-	N.A.	ATRX mut	-	73
LGG 10	M	32	N.A.	+	-	N.A.	ATRX mut	N.A.	23
LGG 11	M	28	N.A.	+	-	N.A.	ATRX mut	N.A.	72
LGG 12	F	59	N.A.	+	-	N.A.	N.A.	Co-deletion 1p/19q	43
LGG 13	M	51	N.A.	-	-	N.A.	-	-	21
LGG 14	M	44	N.A.	+	-	N.A.	N.A.	-	30

Abbreviations: mut, mutated; del, deletion; part del, partial deletion; ampl, amplification; (-), No mutations identified, negative; (+), positive; N.A., Not performed.
